# Access to N-Acetylated Chitohexaose with Well-Defined Degrees of Acetylation

**DOI:** 10.1155/2017/2486515

**Published:** 2017-06-05

**Authors:** Kecheng Li, Ronge Xing, Song Liu, Yukun Qin, Pengcheng Li

**Affiliations:** ^1^Key Laboratory of Experimental Marine Biology, Institute of Oceanology, Chinese Academy of Sciences, Qingdao 266071, China; ^2^Nantong Marine Science and Technology R&D Center, IOCAS, Jiangsu 226006, China

## Abstract

Chitohexaose has attracted wide interest due to its special bioactivities and these potential activities are significantly related to N-acetylation. Herein, six chitohexaose fractions with different degrees of acetylation were prepared by selective N-acetylation and ion-exchange chromatography and further analyzed by ESI/MS. It is revealed that all the six N-acetylated chitohexaoses were of single molecular weight, the molecular weights of which were exactly assigned to 1026.44 Da, 1068.44 Da, 1110.48 Da, 1152.48 Da, 1194.49 Da, and 1236.48 Da, respectively. These results suggested that the six prepared N-acetylated chitohexaoses were N-acetylchitohexaose (D5A1), di-*N*-acetylchitohexaose (D4A2), tri-*N*-acetylchitohexaose (D3A3), tetra-*N*-acetylchitohexaose (D2A4), penta-*N*-acetylchitohexaose (D1A5), and hexa-*N*-acetylchitohexaose (A6), respectively, which are of great significance to screen their bioactivities and discover well-defined chitooligosaccharide molecules as potential drugs.

## 1. Introduction

Chitooligosaccharides, made up of d–glucosamine (GlcN, D) and N-acetyl-d-glucosamine (GlcNAc, A), have been reported to possess various bioactivities including antitumor [1, 2], antimicrobial [3, 4], and immunity modulatory effects [5] and being elicitors of plant defence [6, 7]. These bioactivities are significantly influenced by the molecular size of chitooligosaccharides and their number of N-acetyl groups. At present, chitooligosaccharides can be obtained by chemical or enzymatic hydrolysis of chitin and chitosan [8–10]. Most reported techniques generally produce a complicated heterochitooligosaccharides containing molecules with different molecular weights and degrees of acetylation. It is difficult to determine which molecules are responsible for the observed biological effects. Recently, several single chitooligosaccharides have been obtained by chemical synthesis [11, 12] and chromatographic separation [13, 14]. In particular, of these single chitooligosaccharides, chitohexaose ((GlcN)_6_, D6) is of a great interest due to its special bioactivities. Firstly, chitohexaose has attracted wide attention as a potential antitumor drug. Suzuki et al. reported that chitohexaose displayed remarkable antitumor activity against Sarcoma 180 solid tumors in BALB/C mice as well as against MM-46 solid tumor implanted in C3H/HC mice [15]. Li et al. found that, among the five tested chitooligosaccharides (chitobiose to chitohexaose), chitohexaose had the most potent inhibitory effect on proliferation of the human lung carcinoma cell A549 [16]. Additionally, chitohexaose is also found to be an efficient immunomodulator. Wei et al. reported that chitohexaose could notably promote the secretion of diverse cytokines in vitro and in vivo, including interleukin-1 (IL-1), tumor necrosis factor-alpha (TNF-*α*), and interferon-*γ* (IFN-*γ*) [17]. In agriculture, hexa-*N*-acetylchitohexaose is known as an inducer of disease resistance in crop plants, which could elicit an increase of lignification-related and antioxidative enzymes in soybean plants [18].

The potential activity of chitohexaose was significantly related to N-acetylation. Lieder et al. compared the effect of hexa-*N*-acetylchitohexaose and chitohexaose on short-term expansion and osteogenic differentiation of human mesenchymal stem cells and found that hexa-*N*-acetylchitohexaose had significantly stronger effects than chitohexaose [19]. However, current researches on chitohexaose all focused on the fully acetylated or deacetylated hexamers due to the test sample unavailability of other chitohexaoses with intermediate number of N-acetyl groups. Herein, we reported the straight access to N-acetyl chitohexaose composed of well-defined glucosamine and N-acetylglucosamine unit by selective N-acetylation of chitohexaose and subsequent ion-exchange chromatography separation. These prepared hexamers were of great significance for further study of their bioactivity and action mechanism.

## 2. Experiments

### 2.1. Materials

Chitohexaose hydrochloride (≧98%) was prepared according to the method reported by previous study [20]. CM Sephadex C25 was purchased from GE Healthcare, USA. Darco G-60 activated charcoal (100 mesh) was purchased from Sigma Chemicals Co. Acetic anhydride, phenol, and other chemical reagents were of analytic grade without further purification.

### 2.2. N-Acetylation of Chitohexaose

The selective N-acetylation reaction was carried out according to the method reported by Trombotto et al. [21] with slight modification. Briefly, 200 mg of chitohexaose hydrochloride was dissolved in 10 mL of a methanol/water (50 : 50, v/v) solution. Acetic anhydride (200 *μ*L) was added stoichiometrically to the chitohexaose solution under magnetic stirring at room temperature for 1 h. Subsequently, the resulting solution was concentrated and lyophilized.

On the other hand, 200 mg of chitohexaose hydrochloride was dissolved in 5 mL of a water solution. This solution was adjusted to around pH 9 using 1.0 M NaOH solution in order to obtain chitohexaose without protonated amino group. Subsequently, desalination was performed by activated charcoal extraction. The resulting solution was concentrated to 5 mL and mixed with 5 mL methanol and further used for N-acetylation reaction.

### 2.3. Desalination of Chitohexaose

Chitohexaose solution was stirred with activated charcoal (Darco G-60, 100 mesh) for 30 min. Chitohexaose can be adsorbed onto the activated charcoal. This mixture was filtered to remove the aqueous solution containing NaCl. Subsequently, the chitohexaose adsorbed onto the activated charcoal was desorbed by stirring in 50% aqueous ethanol for 30 min.

### 2.4. Separation of N-Acetylated Chitohexaose

The prepared N-acetylated chitohexaose (200 mg) was dissolved in 5 mL of HAc-NaAc buffer (50 mmol/L, pH 4.8) and then filtered with a microporous membrane (0.45 *μ*m) before injection into a column (2.6 × 50 cm) of CM Sephadex C25 for separation. After loading the sample, the column was first eluted with 2-column volume of HAc-NaAc buffer. Then a gradient elution was carried out using different concentrations of NaCl-HAc buffer (0–2 mol/L) stepwise at a flow rate of 3 mL/min. The eluted solution was collected (5 mL/tube) and monitored by the phenol-sulfuric acid method at 490 nm [22]. Fractions were pooled and desalted by activated charcoal extraction and lyophilized.

### 2.5. Characterization

FT-IR spectra of samples were measured in the range of 4000–400 cm^−1^ regions using a Thermo Scientific Nicolet iS10 FT-IR spectrometer in KBr discs.

The ESI/MS analysis was performed using an amaZon SL ion trap mass spectrometer (Bruker, Germany) equipped with an electrospray-ionization source. All spectra were obtained in the positive-ion mode. Samples of chitohexaoses were prepared in water/acetonitrile (50 : 50, v/v) and infused in the source at a flow of 5 *μ*L/min. The capillary voltage was set to 4500 V and the drying gas temperature was 250°C.

The ESI/MS^2^ experiments were performed by varying the collision-induced dissociation after the *m*/*z* of the interest had been isolated. The collision energy was optimized between 10 and 30 V by fragmentation abundance.

## 3. Result and Discussion

**Figure 1:**
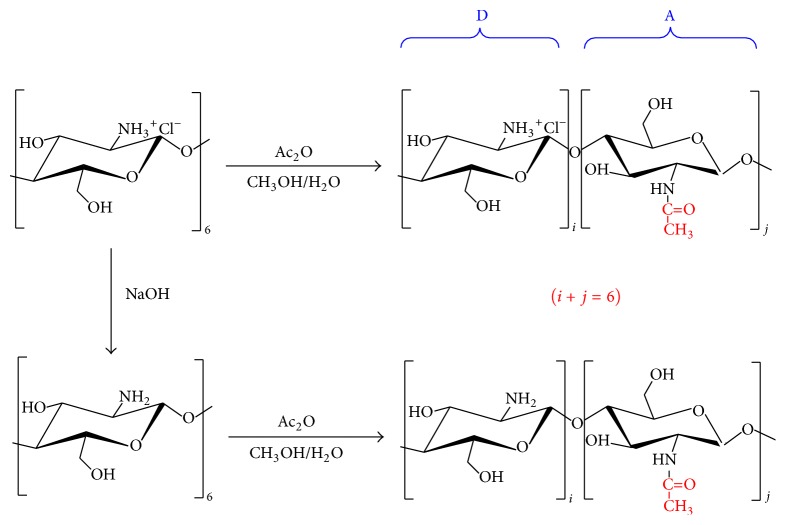
The selective N-acetylation of chitohexaose in two ways.

**Figure 2:**
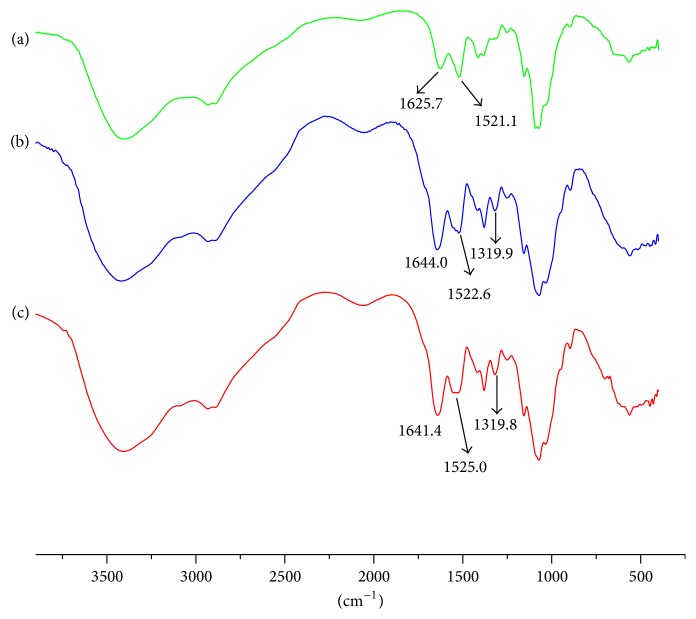
FT-IR spectra of chitohexaose hydrochloride (a), NAD6-1 (b), and NAD6-2 (c).

**Figure 3:**
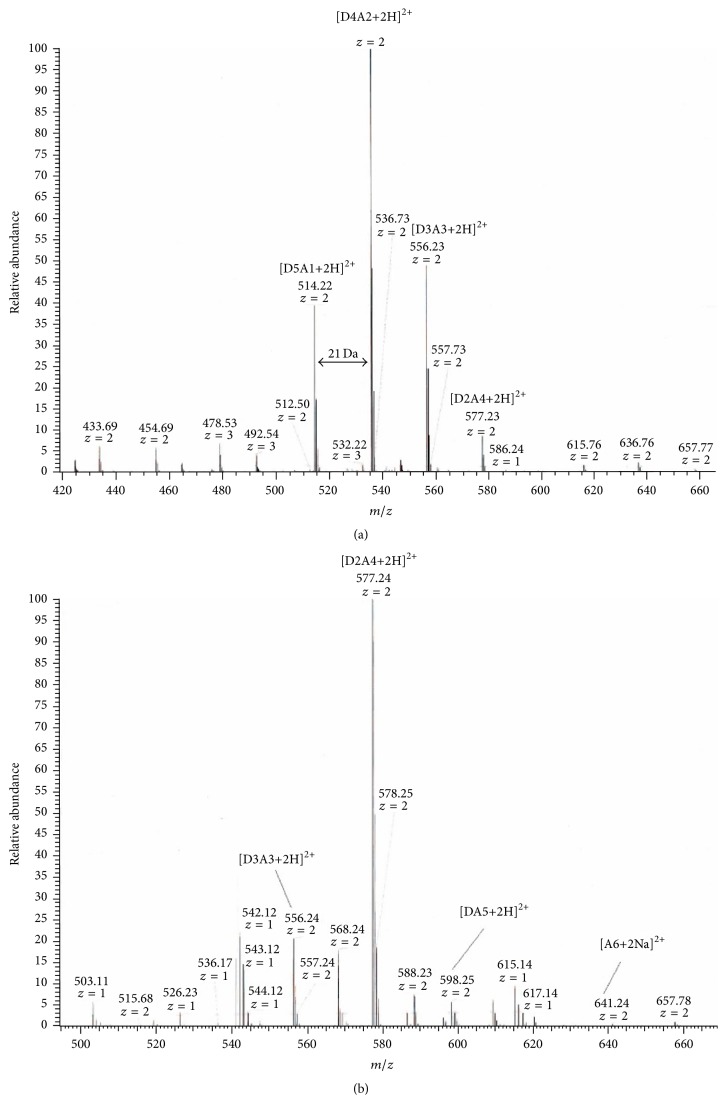
ESI/MS spectra of two prepared chitohexaoses with different degrees of N-acetylation. (a) NAD6-1; (b) NAD6-2.

Considering that –NH_3_^+^ of chitohexaose hydrochloride ((GlcN·HCl)_6_) might reduce the efficiency of N-acetylation, the reaction was performed in two conditions as illustrated in Figure 1. After neutralization, the N-acetylation reaction was performed under the same conditions as before. Two preparations of N-acetylated chitohexaoses, NAD6-1 and NAD6-2, were obtained and further analyzed. N-acetylation reactions were performed at sufficiently soft conditions to avoid the acetylation of hydroxyl groups on the saccharides chains [23], which was confirmed by FT-IR analysis (Figure 2). The bands at 1625 cm^−1^ and 1521 cm^−1^ correspond to the characteristic absorbance peaks of –NH_3_^+^. After acetylation, these bands shifted to 1641~1644 cm^−1^ and 1522~1525 cm^−1^, which were attributed to C=O (amide I) and C–N (amide II) stretching vibration, respectively. In addition, the band at 1319 cm^−1^ (amide III) appeared after acetylation. In particular, in the FT-IR spectra of NAD6-1 and NAD6-2, the band at around 1735 cm^−1^ was not observed after acetylation, which was assigned to the absorbance peak of –COO– [24]. Therefore, the acetylation only occurred on the amino group in our conditions. The ESI/MS ion peaks of the main components of NAD6-1 and NAD6-2 are shown in Figure 3. All of the peaks were [M+2H]^2+^ or [M+2Na]^2+^ ion peaks (e.g., N-acetylchitohexaose, D5A1: calculated mass: [179(C_6_H_13_O_5_N) + 5 × 161(C_6_H_11_O_4_N) + 42(COCH_2_) + 2(H)]/2 = 514 Da). In the ESI/MS spectrum of NAD6-1, the observed *m*/*z* 514.22, *m*/*z* 535.22, *m*/*z* 556.23, and *m*/*z* 577.23 correspond to the ion peaks of N-acetyl chitohexaose (D5A1), di-*N*-acetyl chitohexaose (D4A2), tri-*N*-acetyl chitohexaose (D3A3), and tetra-*N*-acetyl chitohexaose (D2A4), respectively. NAD6-1 mainly contains some chitohexaoses with low degrees of N-acetylation. Similarly, as is depicted in Figure 3(b), NAD6-2 mainly consisted of those chitohexaoses with high degrees of acetylation. Therefore, the efficiency of N-acetylation was obviously promoted after –NH_3_^+^ being changed into –NH_2_. It is proved that the N-acetylation of chitohexaose was susceptible to the form of –NH_2_.

**Figure 4:**
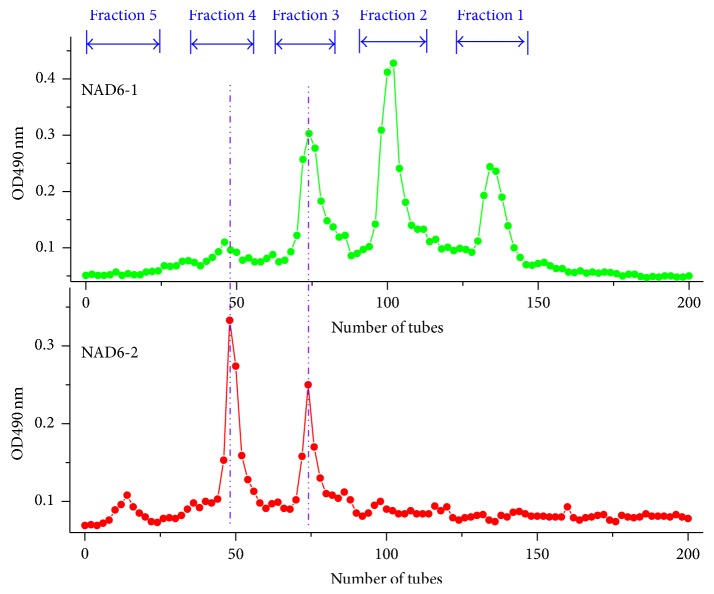
Chromatographic profiles of NAD6-1 and NAD6-2 on CM Sephadex C-25 column.

The prepared N-acetylated chitohexaoses, NAD6-1 and NAD6-2, were further separated using ion-exchange chromatography according to their numbers of amino groups.  Figure 4 describes the chromatographic profiles of NAD6-1 and NAD6-2 on CM Sephadex C-25 column eluted by successively increasing concentrations of NaCl. The retention times of N-acetylated chitohexaoses increase with the decreasing of N-acetylglucosamine unit numbers. Four distinct fractions, corresponding to the N-acetylated chitohexaoses with different composition units, were separated from NAD6-1, while three fractions were isolated from NAD6-2. Compared with NAD6-1, NAD6-2 was eluted by lower concentrations of NaCl, indicating that there indeed exists obvious difference in the degree of N-acetylation between NAD6-1 and NAD6-2 as mentioned above. It is worth noting that some fractions in NAD6-1 and NAD6-2 have almost the same retention time. These fractions are inferred to be those N-acetylated chitohexaoses having identical number of N-acetylglucosamine units and correspondingly combined. Finally, five well-separated fractions (Fractions 1~5) were collected. According to the retention time, it could be predicted that Fractions 1~5 are N-acetylchitohexaose (D5A1), di-*N*-acetylchitohexaose (D4A2), tri-*N*-acetylchitohexaose (D3A3), tetra-*N*-acetylchitohexaose (D2A4), and penta-*N*-acetylchitohexaose (D1A5), respectively. In addition, the neutral oligosaccharide, hexa-*N*-acetylchitohexaose (A6), is uncharged in the buffer and could not be absorbed on the ion-exchange resin. Thus, A6 is concluded to be in the buffer solution (0 mol/L NaCl) at the beginning of elution. This solution was collected and referred to Fraction 6.

**Figure 5:**
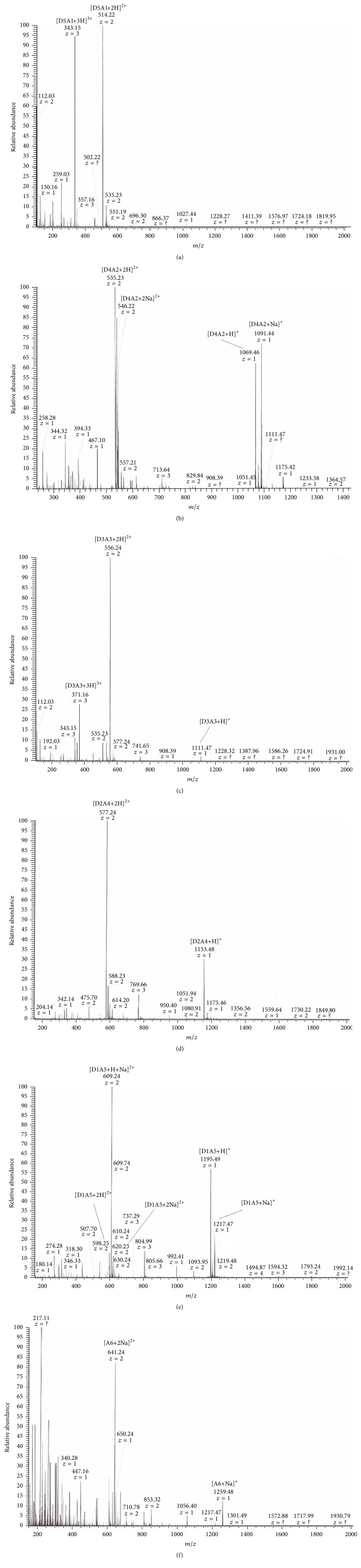
ESI/MS spectra of six separated chitohexaose fractions with different degrees of acetylation. (a~f) Fractions 1~6.

ESI/MS analysis of Fractions 1~6 further proved our prediction about their components (Figure 5). In the positive mode, diverse ion peaks with different *m*/*z* are observed in the ESI/MS spectra, such as [M+H]^+^, [M+Na]^+^, [M+2H]^2+^, [M+2Na]^2+^, [M+2H+Na]^3+^, and [M+3H]^3+^. The six separated fractions are judged to be relatively pure based on the mass spectra. The molecular weights of Fractions 1~6 are exactly assigned to 1026.44 Da (D5A1), 1068.44 Da (D4A2), 1110.48 Da (D3A3), 1152.48 Da (D2A4), 1194.49 Da (D1A5), and 1236.48 Da (A6), respectively. The difference between the mass/charge ratios of adjacent peaks is 42 Da, which is exactly the molecular mass of an acetyl residue.

**Figure 6:**
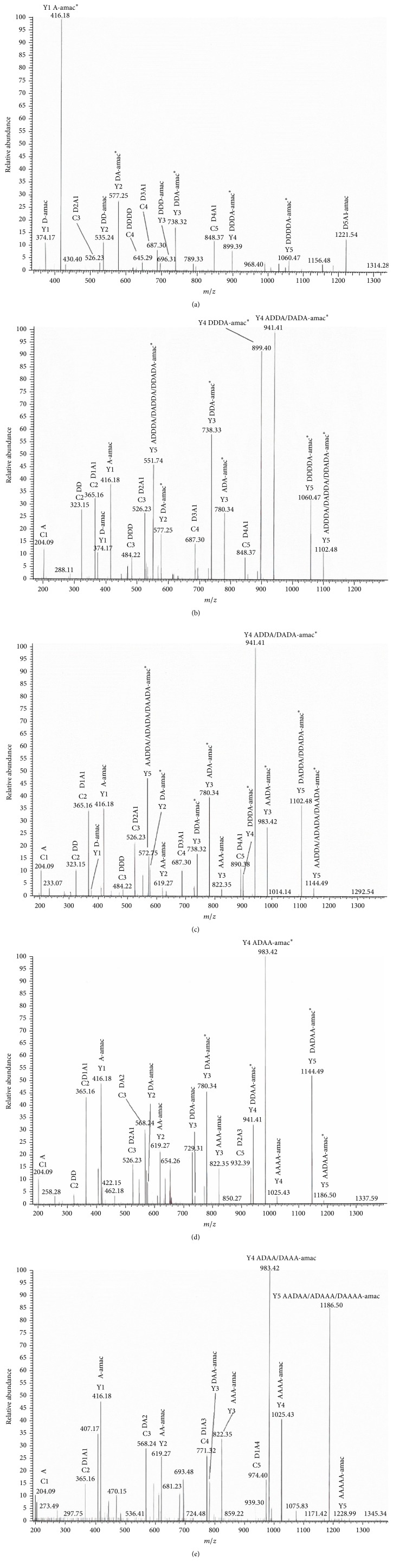
ESI-MS^2^ spectra of five partially N-acetylated chitohexaoses. (a) N-Acetylchitohexaose (D5A1), (b) di-*N*-acetylchitohexaose (D4A2), (c) tri-*N*-acetylchitohexaose (D3A3), (d) tetra-*N*-acetylchitohexaose (D2A4), and (e) penta-*N*-acetylchitohexaose (D1A5). *∗* refers to the main components according to the intensity of ion peaks.

However, the prepared six chitohexaoses are still composed of some possible isomers with various sequences except hexa-*N*-acetylchitohexaose. For instance, N-acetylchitohexaose might contain six isomers, including ADDDDD, DADDDD, DDADDD, DDDADD, DDDDAD, and DDDDDA. In order to identify the main components of these N-acetylated chitohexaoses, sequence analysis was performed by ESI/MS^2^. It is necessary that a tag of 2-aminoacridone (amac) was introduced at the reducing end of the molecule before the MS experiments so as to distinguish a fragmentation of native hetero-chitohexaose from the reducing or nonreducing end [23].  Figure 6(a) illustrates the MS^2^ spectrum of the [M+H]^+^ ion of *m*/*z* 1221 of the derivatized N-acetyl chitohexaose (D5A1). Y-type fragment ion is observed at *m*/*z* 416.18 corresponding to A-amac. The low-intensity peak appearing at *m*/*z* 374.17 indicates a relatively low amount of D-amac. It is suggested that the peak at *m*/*z* 577.25 could be mainly assigned to Y-type ion of DA-amac and partially to Y-type ion of AD-amac. In the same manner, the peaks of *m*/*z* 738.32, 899.39, and 1060.47 mainly correspond to the Y-type ions of DDA-amac, DDDA-amac, and DDDDA-amac, respectively. Therefore, the separated N-acetylchitohexaose is a mixture with several different sequence isomers and mainly contains DDDDDA. It is implied that the glucosamine at the reducing end prefers to be acetylated. This finding is consistent with the result of Tokuyasu et al. [25].

In analogy to the assignment of D5A1 sequences, the analytic results of all the fractions were summarized in Table 1. D4A2 and D3A3 are relatively complicated and possibly consist of more than ten isomers of different sequences. According to the intensity of ion peaks, the isomers of ADDDDA, DADDDA, DDADDA, and DDDADA are the major components of D4A2 and the isomers of ADADDA and ADDADA are superior to other isomers in the product of D3A3. These results indicated that the N-acetylation reaction seldom occurred on the adjacent glucosamine unites, which may result from the steric hindrance. Then with the acetylation reaction going, the number of isomers of N-acetylated chitohexaoses declined but the isomers with N-acetylglucosamine at the reducing end are still the main product. As is shown in Table 1, D2A4 is composed of seven isomers and ADADAA is the major component. D1A5 mainly contains three isomers, AAADAA, AADAAA, and ADAAAA.

## 4. Conclusion

In conclusion, we have developed a process route for the production of N-acetylated chitohexaose with well-defined degrees of acetylation. All the obtained six N-acetylated chitohexaoses were of single molecular weight, including N-acetyl chitohexaose, di-*N*-acetylchitohexaose, tri-*N*-acetylchitohexaose, tetra-*N*-acetylchitohexaose, penta-*N*-acetylchitohexaose, and hexa-*N*-acetylchitohexaose. This method is feasible to prepare other single DP chitooligosaccharides with different degrees of acetylation and these products would be favorable to screen their bioactivities and discover well-defined chitooligosaccharide molecules as potential drugs.

## Figures and Tables

**Table 1:** MS^2^ fragments of six N-acetylated chitohexaoses-AMAC derivatives.

D_*i*_A_*j*_ -amac (*i* + *j* = 6)	Y-ion	*m*/*z*	Fragment	Sequences
D5A1-amac	Y1	374.17	D-amac	DDDDDA^*∗*^, DDDADD, DDDDAD, DDADDD
		416.18	A- amac^*∗*^	
	Y2	535.24	DD-amac	
		577.25	DA-amac^*∗*^, AD-amac	
	Y3	696.31	DDD-amac	
		738.32	DDA-amac^*∗*^, ADD-amac, DAD-amac	
	Y4	899.39	DDDA-amac^*∗*^, DADD-amac, DDAD-amac, ADDD-amac	
	Y5	1060.47	DDDDA-amac^*∗*^, DDADD-amac, DDDAD-amac, DADDD-amac	

D4A2-amac	Y1	374.17	D-amac	ADDDDA^*∗*^, DADDDA^*∗*^, DDADDA^*∗*^, DDDADA^*∗*^, ADDADD, ADDDAD, ADADDD, DADADD, DADDAD, DAADDD, DDAADD, DDADAD, DDDAAD
		416.18	A- amac^*∗*^	
	Y2	535.24	DD-amac	
		577.25	DA-amac^*∗*^, AD-amac	
	Y3	696.31	DDD-amac	
		738.33	DDA-amac^*∗*^, ADD-amac, DAD-amac	
		780.34	ADA-amac^*∗*^, AAD-amac	
	Y4	899.40	DDDA-amac^*∗*^, DADD-amac, DDAD-amac, ADDD-amac	
		941.41	ADDA-amac^*∗*^, AADD-amac, ADAD-amac, DADA-amac^*∗*^, DAAD-amac	
	Y5	1060.47	DDDDA-amac^*∗*^, DDADD-amac, DDDAD-amac, DADDD-amac	
		1102.48 (551.74, *z* = 2)	ADDDA-amac^*∗*^, ADADD-amac, ADDAD-amac, AADDD-amac, DADDA-amac^*∗*^, DAADD-amac, DADAD-amac, DDADA-amac^*∗*^, DDAAD-amac	

D3A3-amac	Y1	374.17	D-amac	ADADDA^*∗*^, ADDADA^*∗*^, ADADAD, ADDAAD, ADDDAA, AADDDA, AADDAD, DAADDA, DAADAD, DADADA, DADAAD, DADDAA, DDAADA, DDAAAD, DDADAA, DDDAAA
		416.18	A- amac^*∗*^	
	Y2	577.25	DA-amac^*∗*^, AD-amac	
		619.27	AA-amac	
	Y3	738.33	DDA-amac^*∗*^, DAD-amac	
		780.34	ADA-amac^*∗*^, AAD-amac, DAA-amac	
		822.35	AAA-amac	
	Y4	899.40	DDDA-amac^*∗*^, DDAD-amac	
		941.41	ADDA-amac^*∗*^, ADAD-amac, DADA-amac^*∗*^, DAAD-amac, DDAA-amac	
		983.42	AADA-amac^*∗*^, AAAD-amac, ADAA-amac, DAAA-amac	
	Y5	1102.48	DADDA-amac^*∗*^, DADAD-amac, DDADA-amac^*∗*^, DDAAD-amac, DDDAA-amac, ADDDA-amac, ADDAD-amac	
		1144.49 (572.75, *z* = 2)	AADDA-amac^*∗*^, AADAD-amac, ADADA-amac^*∗*^, ADAAD-amac, ADDAA-amac, DAADA-amac^*∗*^, DAAAD-amac, DADAA-amac, DDAAA-amac	

D2A4-amac	Y1	416.18	A- amac^*∗*^	ADADAA^*∗*^, AAADDA, AADDAA, ADDAAA, DAADAA, DADAAA, DDAAAA
	Y2	577.25	DA-amac	
		619.27	AA-amac	
	Y3	738.33	DDA-amac	
		780.34	DAA-amac	
		822.35	AAA-amac	
	Y4	941.41	ADDA-amac, DDAA-amac^*∗*^	
		983.42	ADAA-amac^*∗*^, DAAA-amac	
		1025.43	AAAA-amac	
	Y5	1144.49 (572.75, *z* = 2)	AADDA-amac, ADDAA-amac, DADAA-amac^*∗*^, DDAAA-amac	
		1186.50	AADAA-amac^*∗*^, ADAAA-amac, DAAAA-amac	

D1A5-amac	Y1	416.18	A- amac^*∗*^	AAADAA^*∗*^, AADAAA^*∗*^, ADAAAA^*∗*^, DAAAAA
	Y2	619.27	AA-amac	
	Y3	780.34	DAA-amac AAA-amac	
		822.35		
	Y4	983.42	ADAA-amac, DAAA-amac^*∗*^ AAAA-amac	
		1025.43		
	Y5	1186.50 (593.75, *z* = 2)	AADAA-amac^*∗*^, ADAAA-amac, DAAAA-amac	
		1228.99	AAAAA-amac	

*∗* means being judged as the main components according to the intensity of ion peaks.
